# Effects of storage bags type and storage duration on seed quality and proximate composition of emmer wheat (*Triticum dicoccum L.*) in Ethiopia

**DOI:** 10.1016/j.heliyon.2022.e12506

**Published:** 2022-12-22

**Authors:** Bethlehem Melese, Neela Satheesh, Solomon Workneh Fanta, Zewdie Bishaw

**Affiliations:** a Faculty of Chemical and Food Engineering, Bahir Dar Institute of Technology, Bahir Dar University, Bahir Dar, Ethiopia; b Seed Technology, Wondogenet Research Center, Ethiopian Institute of Agricultural Research, Wondogenet, Ethiopia; c International Center for Agricultural Research in Dry Areas, Addis Ababa, Ethiopia; d Department of Food Nutrition and Dietetics, Faculty of Agriculture, Sri Sri University, Cuttack, Odisha, India

**Keywords:** PICS bags, GrainPro bags, Emmer wheat, Filter cake, Triplex, Seed germination, Storage durations, Protein, Polypropylene bags

## Abstract

This study was conducted to evaluate the effect of storage materials (Storage bags) and durations on seed quality and proximate composition of emmer wheat in farmer storage practices. The emmer wheat samples were stored for nine consecutive months in PICS (Purdue Improved Crop Storage) bag (PB), Grainpro® Bag (GPB), Polypropylene Bag (PPB), emmer wheat treated with Filter Cake (a byproduct of Aluminum Sulphate factory) (FC), stored in Polypropylene bag (PPBFC) and Emmer wheat treated with triplex (a by-product of soap factory) (TX) stored in Polypropylene bag (PPBTX). Data on seed quality and proximate composition were evaluated every three months’ interval for 9 months. As storage period increased from three to nine months; Germination Percentage, Speed of Germination, Vigour, Thousand Seed Weight (TSW), Bulk Density (BD), Seed Damage%, Seed Weight Loss, Protein and Carbohydrate contents were significantly influenced by the interaction effect of storage period and storage bag used. The highest germination (98%) was recorded from seeds stored in GPB for three months. The protein content of grains stored in GPB for three months showed the highest (8.3%) whereas, the lowest (6.5%) was for PPB at nine months of storage. Minimal insect incidence and lower seed weight loss were observed in emmer wheat stored in bags such as BP, GPB, PPBFC, and PPBTX. The use of PB and GPB, as well as the application of FC and TX maintained the proximate and seed quality of emmer wheat.

## Highlights

• Highest germination percentage (98 %) of emmer wheat was reported for seed stored in Grainpro bags.
• The higher (5001 mg%) vigour index was recorded from seeds treated with triplex and stored in polypropylene bag.
• Higher rate of seed damage (5%) and seed weight loss (3.9%) was reported for seeds stored in polypropylene bags.
• Emmer wheat seeds stored in Grainpro bags had showed the highest (78%) carbohydrate content.
• Seeds treated with filter cake and triplex had showed the highest (2%) ash content.

## 1 Introduction

Emmer wheat is one of the most important cereal crops of Ethiopia after teff, maize, wheat and sorghum. It is cultivated in about 24000 ha of agriculture land, with production of 49,200 tons in 2019 [1]. Emmer wheat production is very low compared to other cereal crops in the country due to several constraints including the availability of limited number of improved varieties, limited availability and poor quality of seeds, substandard cultural practices and deprived postharvest storage practices [2].

Kaske et al. [4], reported pests like granary weevil (*Sitophilus granarius* (L.) (*Coleoptera: Curculionidae*)); Lesser grain borer (*Rhyzopertha dominica* (F.) (*Coleoptera: Bostrichidae*)); rice weevil (*Sitophilus oryzae* (L.) (*Coleoptera: Curculionidae*)); and maize weevil (*Sitophilus zeamais Motschulsky (Coleoptera: Curculionidae*)) are the most common pests occurred in Ethiopian wheat storage. Greater variation was reported by previous authors in the magnitude of grain loss by the storage pests in the Ethiopia. For instance, Marid & Md Jamshed [3], estimated about 10–30% of the typical cereal grain losses due to the storage insect pests, in contrast, Kaske et al. [4] reported that the mean loss of wheat in Ethiopia is attributed to the storage insects as 1.5%.

Seed is a crucial component that has a significant impact on crop output and productivity. Higher quality seed is regarded as the most important and basic input for boosting productivity and increasing net monetary rewards per unit area [5]. Approximately, 90% of Ethiopia's farmed wheat land is sown using seeds sourced locally [6] usually, seeds saved by farmers from previous season harvest [7]. Ethiopian farmers store seeds traditionally under poor condition (packed in polypropylene bags in the house or store in gotera/gota (a mud construction) without packing in any bag). Poorly handled seeds in storage performs poorly in the field, diminishing yields and affecting food security [8].

Improper storage conditions lowers the seed germination capacity, seed quality degradation, and loss of potentiality [9]. Physiological quality of the seeds is influenced by storage temperature, humidity, chemical effects, production settings, harvesting, illnesses, and insects during seed storage. Furthermore, storage environment and packing materials have substantial impact on seed quality and proximate composition [10]. Duration of the storage and storage conditions also may induce some physical, chemical and biochemical alterations in seed [11] and influencing the grain quality. Prolonged storage of wheat seed also causes reduction in seed germination, seedling vigour, and increases in germination time, insect infestation and finally, loss in seed weight [13]. The germ damage of the seed also influenced by the storage conditions, further, germ effects drastically and leads to the viability loss [13]. Hence, to retain seed quality, seed should be properly stored in decent storage facilities [14]. Furthermore, proper storage of the food grains is very important to retain the nutritional quality and to maintain the suitability to the consumption [4]. Poor storage conditions significantly decrease proximate composition, nutritional composition of grains and yields poor quality products (like flours) [5]. However, there are many advanced grain storage techniques practicing in the contemporary world, the usage of those technologies in under developed countries like in Ethiopia is technically and economically challenging [4].

Hence, using of cost effective hermetic technologies (PB, GPB and Metal silos) and applying of inert dusts and botanicals at farmers’ storage practices as alternate storage solutions to protect the quality of stored grains has been documented in previous studies [14, 15]. Inert dusts capacity as alternative to the synthetic pesticides has been established in previous study [16]. Similarly, Kalsa et al. [17], documented the pesticide activity of FC dust against *S. granarius* and *R. dominica.*

Almost all Ethiopian farmers store their seeds in indigenous storage materials ('*Gotera'/'Gota*,' and PPB) in their home [18]. In addition, they also apply synthetic insecticides to protect insect infestation in stored grains [21]. For postharvest preservation of cereals like maize, previous study [19] reported the application of FC, TX, and usage of metal silos in Ethiopia. Only three research reports [17, 19, 20] are available on FC and TX on preservation of wheat.

Despite various studies are reported on the use of alternative storage procedures to avoid seed loss and to maintain grain quality, best of our knowledge very limited information scientifically available on storage of emmer wheat at farmers practice in Ethiopia. Furthermore, the comparative performance evaluation of various cost effective emmer wheat preservation systems has not been studied. The findings of the present study directly support the emmer wheat growing farmer community to extended storage of emmer wheat in the study location. Hence, the major objective of this study was to examine the effect of different storage materials and time frames on emmer wheat seed quality and proximate composition.

## 2 Materials and methods

### 2.1 Emmer wheat sample collection

Eleven quintals of local landrace (most widely cultivated variety by Ethiopian farmers) emmer wheat sample was acquired from Sinana Agricultural Research Center. The seed was harvested in May 2021 and had not been treated with any pesticides or preservatives. The collected sample was manually cleaned to remove plant debris, dust, stones and extraneous materials.

### 2.2 Description of experimental storage bags

The PPB of 50 kg capacity were bought from the local market. The inert dust samples, FC obtained from Awash Melkassa Aluminum Sulphate & Sulphuric Acid Share Company and TX was procured from Star Soap and Detergent Industries, Ethiopia. The GPB (produced by Grainpro Company, Concord, Massachusetts, USA) of 100 kg capacity were obtained from HiTEC Trading PLC, Addis Ababa, Ethiopia. This bag is made up of a single layer of 78 μm plastic film extremely impermeable to oxygen [22]. The PB is made up of one woven polypropylene bag wrapped by two layers of 80 mm thick high-density polyethylene (HDPE) procured from local authorized supplier [23].

### 2.3 Experimental design and setup

This study was conducted for 9 months of duration (from June 28, 2021 to February 28, 2021) under farmers' storage conditions. The experiment consisted of two factors arranged in CRD. The first factor considered was storage bag consisted of five types i.e. PB, GPB, PPB, PPBFC and PPBTX. The second factor is a storage period consisted of four levels (Initial day followed by three, six and ninth months of storage durations). Each storage bag was filled with cleaned 25 kg of emmer wheat samples, closed tightly and stored in famers’ seed storage place. The inert dust, TX was applied at 0.25% (W/W), while FC was applied at 1% (W/W) as recommended by previous research [20]. Both the TX and FC were applied once to the emmer wheat samples at the beginning of the experiment. All treatments were analyzed in triplicate and the total runs conducted in this experiment were 60.

The average relative humidity and temperature of the room was recorded daily using HOBO (HOBO MX1101, onset computer Corp., Bourne, Massachusetts, USA) portable temperature/humidity data logger over the storage periods.

### 2.4 Emmer wheat sampling from storage bags

Before storage experiment was commenced, baseline data of the emmer wheat sample was collected. During the 9 months’ storage, samples were taken at every three-month interval. Hand sampling method was followed to collect around 5-kilogram sample from each storage run by following the guidelines and sampling plans provided by the ISTA (2014) [24]. Further, collected sample was properly mixed and abridged to one kilogram for seed quality and proximate composition analysis.

### 2.5 Data collection

The following seed quality and proximate compositions parameters of emmer wheat samples were determined.

#### 2.5.1 Seed quality analysis

##### 2.5.1.1 Germination percentage

Germination percentage was determined accordance to the ISTA (2014) [24] guidelines. In four replications, 200 seeds were randomly selected from each sample and placed in a separate germination box. The germination chambers were used for this test and conducted for eight days to observe the seed germination. The following Eq. (1) was used to compute the germination percentage.

(1) $$Germination\;\left(\%\right)=\frac{Number\;of\;normal\;seedling}{Total\;number\;of\;seeds\;sown\;}\;x\;100\ldots \ldots$$

##### 2.5.1.2 Speed of germination

The speed of germination was assessed by using the similar method of germination percentage determined, except that the number of germinated seeds was tallied and removed every day until no more germination occurred [24]. The speed of germination was calculated using Eq. (2).

(2) $$Speed\;of\;germination=\frac{N1}{C1}+\frac{N2}{C2}+\ldots +\frac{NF}{CF}\ldots \ldots$$

Where: N1, N2, NF is the 1^st^, 2^nd^ and final day counts of germinated normal seedlings and C1, C2, CF is 1^st^, 2^nd^ and final count of seedlings.

##### 2.5.1.3 Seedling vigour index

During the regular germination test, 10 normal seedlings were selected, placed in an envelope and oven dried at 80 °C for 24 h and cooled down for 30 min in silica gel [25]. The dry weight of all the seedlings was determined by electronic balance with an accuracy of ±0.1 mg. The dry weight of the single seedling was calculated by dividing the dry weight with ten. The seedling vigour index was calculated by multiplying the percentage of kernels that germinated by the mean dry weight (mg) of a single seedling.

##### 2.5.1.4 Thousand seed weight

Using an INDOSAW Digital Seed Counter, a thousand kernels from each samples were counted and weight was determined as the TSW [26].

##### 2.5.1.5 Bulk density (BD)

Emmer wheat samples BD was determined according to the procedure of Chowdhury et al, [26] by the help of standard BD measuring unit of 0.5 L capacity. After, the weight of the seeds was determined by digital electronic balance with the accuracy of ±0.1 g. The following Eq. (3) was used to compute the BD for each replication:

(3) $$Bulk\;density\;\left(\frac{Kg}{m}\right)=\frac{Weight\;of\;the\;sample\;\left(Kg\right)}{Volume\;of\;the\;sample\;\left(m^{3}\right)}\ldots \ldots$$

##### 2.5.1.6 Percentage seed damaged

Exactly one kilogram of emmer wheat seed was sampled from each storage run. The seeds damaged and healthy were separated, counted and weight was noted separately in sub-samples of 100g [30]. Eq. (4) was used to compute the proportion of insect-damaged seed:

(4) $$Insect\;damaged\;seed\;\left(\%\right)=\frac{Number\;of\;insect\;damaged\;seed}{Total\;number\;of\;seed}X\;100\ldots \ldots$$

##### 2.5.1.7 Seed weight loss

The count and weight method proposed by the Boxall [28] was used to determine the weight loss percentage with the help of following Eq. (5).

(5) $$Weight\;loss\;\left(\%\right)=\frac{\left(\;Wt.\;Healthy\;seed*Num.\;Healthy\right)-\left(Wt.\;Damaged\;seed\;*Num.Damaged\;seed\right)}{Wt.\;Undamaged\;seed\;\left(Num.\;damaged+Num.\;Undamaged\right)}X\;100\ldots$$

#### 2.5.2 Proximate composition analysis

The moisture content was determined by using the hot air oven technique (AOAC [29], method number 925.10) gravimetrically. With a conversion factor of 5.7, the protein content of samples was determined using the Kjeldhal technique (AOAC [29], method number 979.09). Fat content was determined using the soxhelet apparatus by using the hexane as the extraction solvent. The ash content was determined by the incineration of sample in muffle furnace (AOAC [29], method number 923.03), fibre content was determined using the non-enzymatic gravimetric method [29]), and carbohydrate content was determined using the difference method [30].

#### 2.5.3 Storage environment during the experiment

The average storage temperature during the experiment was ranged from 11 to 18 °C while, the average relative humidity ranged from 41 to 60 % in the study duration.

### 2.6 Data analysis

All the data obtained in this study was statistically analyzed. The SAS version 9.3 was used to perform the Analysis of Variance (ANOVA). Tukey's test was used to identify significant differences between treatment means at a 5% level of significance. Before doing an analysis of variance, the data on percent seed damage and weight losses were log transformed. Multiple regression analysis was performed to determine the relation between seed germination and seed vigour index and bulk density.

## 3 Results and discussions

ANOVA result showed that the interaction effects of bag types used and storage durations had significantly (P < 0.01) influenced germination %, speed of germination, vigour index, bulk density, thousand seed weight, percentage damage, weight loss, moisture content, protein, and carbohydrate content whereas, fat, fiber and ash contents are not influenced by the interaction effect of factors. The main effects of all factors had showed significant influence in all the studied parameters (Table 1).

**Table 1:** ANOVA p-values for main and interaction effects of storage bags (A) and storage duration (B) on seed quality and proximate composition of emmer wheat.

Term	Germination%	Speed of germination	Vigour index (mg%)	TSW (g)	Bulk Density (kg/m^3^)	Seed damaged (%)	Weight loss (%)	Moisture content (%)	Protein (%)	Carbohydrate (%)	Ash (%)	Fat (%)	Fiber (%)
A	0.001∗	0.000∗	0.000∗	0.000∗	0.000∗	0.000∗	0.000∗	0.000∗	0.000∗	0.000∗	0.001∗	0.1^ns^	0.000∗
B	0.001∗	0.01∗	0.000∗	0.000∗	0.000∗	0.000∗	0.000∗	0.000∗	0.000∗	0.000∗	0.000∗	0.1^ns^	0.000∗
A∗B	0.001∗	0.000∗	0.000∗	0.000∗	0.000∗	0.000∗	0.000∗	0.000∗	0.000∗	0.000∗	0.06^ns^	0.17^ns^	0.1^ns^
R-square	0.98	0.80	0.984	0.996	0.995	0.992	0.997	0.941	0.979	0.917	0.687	0.766	0.991

Note:∗ = significant at 5 % probability level and ns = non-significant.

### 3.1 Effect of storage bags, and duration on seed quality of emmer wheat

#### 3.1.1 Seed germination percentage

Germination is the prime important property of a seed and it indicates the viability [31]. In this study it was understand that, storage materials used were showed effect on emmer wheat seed germination capacity during the studied storage duration [35]. The overall germination rate of seeds was around 98 percent at the onset of trial. The results revealed that the interaction impact of storage materials and storage duration had significant (P < 0.01) influence on the germination percentage of emmer wheat seed (Table 2). Seeds stored in GPB retained the highest germination for longer durations than samples stored in PB and PPE. With increase of storage period from three to nine months, germination percentage of seeds stored in PPB decreased significantly (Table 2). The least germination (72.5%) was reported for seeds stored in PPB for 9 months without any treatments. The lower germination rate of seeds stored in PPB is associated with the availability of moisture and air movement inside and within the PPB and surrounding environment. At this environmental conditions insect growth increases further, the insects feed on the grains and results in damage of emmer wheat germ.

**Table 2:** Seed quality of emmer wheat as affected by stored in different bags for nine months.

Storage bags	Storage periods	Seed quality				
		Germination (%)	Speed of germination	Vigour index (mg %)	Thousand seed weight (g)	Seed bulk density (kg/m^3^)
PPE	Initial day	98^a^	29^a^	4195^a^	60.2^a^	123^a^
	Month 3	97^ab^	30.8^bcd^	3117^n^	54^c^	121.5^a^
	Month 6	86.5^ef^	30.1^bcd^	3252^k^	42.7^d^	115.5^cd^
	Month 9	72.5^gh^	35^a^	2740^q^	32^g^	102^f^
PB	Initial day	98^a^	29^a^	4195^a^	60.2^a^	123^a^
	Month 3	95.5^abc^	31^bcd^	3084°	59.5^a^	122.5^a^
	Month 6	88.5^def^	29^d^	3309j	43^e^	118^bc^
	Month 9	80.5^f^	34.5^a^	2778^p^	39^f^	103^f^
GPB	Initial day	98^a^	29^a^	4195^a^	60.2^a^	123^a^
	Month 3	98^a^	30.5^bcd^	4193^a^	60^a^	120.5^ab^
	Month 6	91^bc^	29.4^bcd^	3713^e^	57^b^	114^de^
	Month 9	87^ef^	30.1^bcd^	3590^h^	53^d^	104.5^f^
PPBFC (0.25% (w/w))	Initial day	98^a^	29^a^	4195^a^	60.2^a^	123^a^
	Month 3	96^a^	30.7^a^	3925^b^	54^b^	119.5^ab^
	Month 6	95^ab^	29.3^bc^	3710^cd^	48^bc^	113^de^
	Month 9	94^abc^	30.1^ab^	3840^bc^	43.2^cd^	104^f^
PPBTX (1% (w/w))	Initial day	98^a^	29^a^	4195^a^	60.2^a^	123^a^
	Month 3	95^abc^	28.1^bc^	5001^a^	40^de^	118^bc^
	Month 6	93.5^bcd^	29.4^ab^	3941^b^	44^cd^	112^e^
	Month 9	90^ef^	27.5^bcd^	3788^cd^	37^f^	98^g^
CV (%)		1.1	3	2.1	0.9	0.7
*P*-value	Storage period	<.0001	0.0003	<.0001	<.0001	<.0001
	Storage materials	<.0001	0.0193	<.0001	<.0001	<.0001
	Storage period ∗ storage materials	0.002	0.003	<.0001	<.0001	<.0001

Means followed by the same letters within column are not significantly different (*P* > 0.05) by Tukey's test.

Vales are the mean of three observations; PPB = Polypropylene bag; PB = PICS bag; GPB = Grainpro bag; PPBFC = Emmer wheat treated with 0.25% (w/w) filter cake and stored in Polypropylene bags; PPBTX = Emmer wheat treated with 1% (w/w) triplex and stored in Polypropylene bags.

Similarly, Afzal et al. [14], reported wheat seed germination capacity was maintained well in hermetic bags but showed quick decline in seeds stored in PPB. Afzal et al (2019) [14] also mentioned that there is an inverse relation between moisture content and shelf life of grains in the storage. Also concluded that one percentage of moisture increase in the stored grain leads to around half reduction in shelf life. In the same study mentioned that, samples stored in GPB was showed better germination capacities and this was attributed to the limited permeable capacity of the water vapors and oxygen of the bags and this conditions cannot support the development of mold and insect growth [33]. The better germination of seeds stored in GPB implies that GPB are able to maintained seed viability through the storage period. Additionally, previous researcher mentioned that wheat seed stored in PPB showed the lowest germination percentage as compared to seeds stored in PICS bag [48]. Seeds treated with FC and TX had showed comparable germination capacity with seeds stored in PB and GPB and better than that of seeds stored in untreated PPB. Similar to the findings of present study, Kaske et al. [8] report that wheat samples treated with FC and TX had showed good germination capacity than seeds stored in PPB.

### 3.2 Speed of germination

Germination speed of emmer wheat samples were significantly (P ≤ 0.01) influenced by the combined effect of storage bags and storage durations considered in the study (Table 2). High speed of germination was observed for the emmer wheat samples stored in PPBTX for 9 months followed by seeds stored in PPBFC for 6 months. Seeds stored in PPB for 9 months of duration was recorded lower germination speed. Therefore, emmer wheat may loss its germination speed if stored in PPB for prolonged duration beyond nine months. The decrease in the speed of germination may be attributed to the damage of seeds by storage insects. Abebaw et al. [34], also reported that germination speed of teff was significantly influenced by prolonged storage periods. On the contrary, Seadh et al. [35], reported that the interaction effect of storage periods and materials did not have significant influence on speed of wheat germination. The result of this study showed that seeds from the PPBFC and PPBTX had showed better germination speed and there is no statistical difference between seeds from PPBFC and PPBTX in their germination speed. Whereas, seeds stored in PPB showed the slowest germination as the storage period increase (Table 2). It can be speculated that seeds treated with FC and TX showed the better germination speed, this can be attributed to the lower rate of seed damage and weight loss that helps to maintain the viability of the seed.

#### 3.2.1 Vigour index

The ANOVA showed that there was a significantly (P ≤ 0.01) high reduction in seedling vigour index as the storage period increased (Table 2). Seedling vigour index of the emmer wheat samples at the beginning of the study was 4195 mg% however, substantial changes were determined in seedlings vigour between samples stored in different runs in nine months of storage. The interaction effect of storage materials and durations had significantly (*P* ≤ 0.01) influenced vigour of emmer wheat samples studied. The highest (5001 mg%) vigour index was obtained from seeds from the treatment PPBTX for three months while, the lowest (2740 mg%) vigour index was observed for seeds stored in PPB stored for nine months. Seedling vigour index of emmer wheat samples from PPBTX, PPBFC are in comparable with that of samples stored in PB and GPB.

Vigour index was decreased gradually up to nine months of storage in PPB may be attributed to the loss in vigour of seeds during extended storage durations. The low vigour index of seeds in this study could be attributed to the oxidation of nutrient reserve in the endosperm through aging which resulted in significant reduction in the seed capacity to germinate and emerge. The findings of the present study is in harmony with study reported by Rahouma [11], mentioned that the interaction effect of storage duration and materials had a significant effect on vigour index of barley seeds. Similarly, the study conducted by Afzal et al. [14], reported that wheat samples stored in PPB reduced its viability and vigour index as compared with hermetic bags (GPB). From this study, it can be hypothesized that the higher vigour index of emmer wheat from PPBTX may be due to the lower seed damage.

#### 3.2.2 Thousand seed weight

The ANOVA showed that the interaction effect of storage periods and materials had significantly (P ≤ 0.01) influenced TSW of emmer wheat (Table 2). The significant difference was observed in TSW among the emmer wheat stored in different storage materials and storage periods. The TSW was decreased with the increase in storage durations. This occurs probably due to the damage of seeds that causes the weight of the seeds to decrease. The highest TSW (60 g) was recorded for seeds stored in GPB for three months; whereas the lowest (32 g) was for seed stored in PPB for 9 months. Baributsa et al. [36] also reported that the lower (26.2 g) TSW of maize samples recorded after seven months of storage in PPB bag due to the highest level of insect infestations of seeds. In contrast, in the same study samples stored in PB reported higher TSW (30.8 g). In addition, Rahouma [11] also reported that, increasing in storage durations up to six months significantly decreased TSW of barley stored in PPB.

#### 3.2.3 Bulk density

The BD of the emmer wheat samples at the onset of the experiment was 123 kg/m^3^. The BD of seed had decreased continuously as storage periods increased in case of all the storage runs. In case of nine months’ storage, the seeds stored in PPB, PB and GPB showed bulk densities ranging from 122.5 kg/m^3^ to 103 kg/m^3^ (Table 2). The decrease in BD from these packing materials during the study periods ranged from 4.1% - 7.6%. The BD of emmer wheat samples from PPBFC, and PPBTX showed the lowest to all other storage materials up to the nine months of storage. The BD reduction rate was observed high (17.1%) in PPB while, it was 15.5% in GPB at 9 months of storage. Emmer wheat samples from PPBFC, and PPBTX showed 16%–20.4% reduction in BD. Similarly, Kaske et al. [8] found that wheat stored for six months showed a decrease in BD for all storage treatments as storage periods increased. In this study, the BD of the samples from PPBFC, and PPBTX showed lower than the other studied storage treatments. Tadesse et al. [16], reported that, samples treated with the inert dust like FC, TX reported reduction in BD due to the reduced mobility of the seed mass.

#### 3.2.4 Seed damage% and weight loss%

This study results showed that, there was a substantial variation in seed damage% among the storage bags used throughout the storage duration (Table 3). During the initial three months of storages, no significant difference in seed damage% was observed between storage materials used (Table 3). However, emmer wheat samples stored in PPB showed significantly higher rates of seed damage (5%) after 9 months of storage period. Live insect (*Rhyzopertha dominica*) population showed a continuous increase during the studied storage periods. The surge in moisture content of stored grain samples leads to increase in insect population; insects are one of the major reasons for seed weight loss [4]. In this study, the samples from PPBFC, PPBTX showed a significant control of *R*. *dominica* development even at 9 months of storage. A similar study conducted by Kaske et al [8] reported that the application of FC and TX maintained the living mature population of *R*. *dominica* at an insignificant level during the six months storage of wheat.

**Table 3:** Impact of storage bag and periods on percentage seed damage and weight loss of emmer wheat stored for 9 months.

Storage materials	Storage periods	Seed quality	
		Seed damage (%)	Percent weight loss (%)
PPE	Month 3	0.2 ± 0.1^c^	0.1 ± 0.00d^e^
	Month 6	0.8 ± 0.3^b^	0.2 ± 0.00^d^
	Month 9	5 ± 1.3^a^	3.9 ± 0.1^a^
PB	Month 3	0.2 ± 0.00^c^	0.3 ± 0.2^d^
	Month 6	0.2 ± 0.00^c^	0.8 ± 0.3^c^
	Month 9	0.3 ± 0.1^c^	1.2 ± 0.5^b^
GPB	Month 3	0.2 ± 0.00^c^	0.2 ± 0.00^d^
	Month 6	0.2 ± 0.00^c^	0.3 ± 0.2^d^
	Month 9	0.3 ± 0.2^c^	1.4 ± 0.6^b^
PPEFC (0.25% (w/w))	Month 3	0.1 ± 0.1^c^	0.2 ± 0.1^d^
	Month 6	0.2 ± 0.1^c^	0.7 ± 0.2^c^
	Month 9	0.2 ± 0.1^c^	0.3 ± 0.2^d^
PPETX (1% (w/w))	Month 3	0.1 ± 0.1^c^	0.3 ± 0.2^d^
	Month 6	0.1 ± 0.1^c^	0.6 ± 0.1^c^
	Month 9	0.2 ± 0.1^c^	0.2 ± 0.3^d^
*CV* (%)		0.12	0.5
P-value	Storage period	<.0001	<.0001
	Storage material	<.0001	<.0001
	Storage period ∗ storage materials	<.0001	<.0001

Means followed by the same letters within column are not significantly different (P > 0.05) by Tukey's test.

Vales are presented as Mean ± Standard deviation of three observations; PPB = Polypropylene bag; PB = PICS bag; GPB = Grainpro bag; PPBFC = Emmer wheat treated with 0.25% (w/w) filter cake and stored in Polypropylene bags; PPBTX = Emmer wheat treated with 1% (w/w) triplex and stored in Polypropylene bags.

The effectiveness of hermetic technologies (PB and GPB) against insects were well documented by different authors [37, 38]. In present study, the number of *R. dominica* sustained at very lower number in emmer wheat samples stored in hermetic bags for 9 months as compared with seed stored in PPB. This trend may be attributed to the hermetic nature of the PB, the reduced levels of the O_2_ and increasing the accumulation of CO_2_ levels hinders the growth of insects and affects their feeding process [39].

The emmer wheat samples weight loss% significantly influenced by the interaction effect of storage bags used and storage durations considered in the present study (Table 3). Highest (3.9 %) seed weight loss was observed in samples from the PPB stored for 9 months. Application of FC and TX dusts and use of hermetic bags (PB and GP) had showed the lowest rate of seed weight loss as compared to seeds stored in PPB. Similar to the finding of the present study, Chigoverah & Mvumi [40], reported that hermetic storage methods are superior to non-hermetic treatments in supressing the maize seed damage and limiting weight loss during the storage.

#### 3.2.5 Multiple regression analysis between germination, seed vigour and bulk density

Regression analysis between storage period and seed quality (germination, vigour and bulk density) showed high fitness of model with R^2^ values of 0.95, 0.91 and 0.88 respectively. Seed storage period shows 95% variation in germination, 91% in vigour and 88% in bulk density (Table 4). Similarly, previous study [49] reported that, simple linear regression of seed age on germination and seed viability shows high fitness of model.

**Table 4:** Multiple regression analysis between seed storage period and seed quality (Germination, Vigour index and bulk density).

Parameter	MR	R^2^	T	Partial correlation
Germination	1.06	0.95	4.46	0.95
Vigour	1.02	0.91	4.28	0.91
Bulk density	0.99	0.88	4.14	0.88

MR = Multiple regression; R^2^ = Coefficient of determination; T = ‘t’ value statistics.

### 3.3 Effect of storage bags and durations on proximate composition of emmer wheat

The interaction effect of storage bags and durations had significant (P ≤ 0.01) influence on moisture content, protein and carbohydrate content whereas, the main effect of storage periods and materials used had showed significant effect on fiber and ash content of emmer wheat samples.

#### 3.3.1 Moisture content

The moisture content in the emmer wheat samples is the most critical parameter in determining the shelf life of seeds. Seed degeneration is accelerated by higher moisture contents and it leads to the poor seed quality. There was a higher significant (*P* < 0.01) interaction impact of storage materials used and storage periods considered were observed on the moisture content of stored emmer wheat (Table 5). The samples stored in GPB, followed by PB, had the lowest seed moisture during the storage of all the treatments. While the samples stored in the PPB reported the greatest seed moisture content. By the 9 months of storage, the emmer wheat stored in GPB recorded 11% seed moisture followed by PICS bag (11.5%) and highest (12.8%) was observed in samples from PPB. Improved (hermetic) seed storage bags like PB, GPB had recorded the lowest seed moisture contents throughout the storage period in comparison to the grains stored in PPB.

**Table 5:** Moisture (%), protein (%) and carbohydrate (%) content of emmer wheat as affected by interaction effect of storage period and storage bags used.

Storage materials	Storage periods	Moisture Content (%)	Protein Content (%)	Carbohydrate (%)
PPB	Initial day	11.5^bc^	8.5^a^	79^a^
	Month 3	11.8^bc^	8.2^ab^	70.6^bc^
	Month 6	11.7^bc^	7.9^bc^	68^cd^
	Month 9	12.8^a^	6.5^d^	62^f^
PB	Initial day	11.5^bc^	8.5^a^	79^a^
	Month 3	11.9^abc^	8.2^ab^	72.3^b^
	Month 6	11.6^bc^	8^abc^	68.2^cde^
	Month 9	11.5^bc^	7.6^c^	67^de^
GPB	Initial day	11.5^bc^	8.5^a^	79^a^
	Month 3	11.9^abc^	8.3^a^	78^a^
	Month 6	11.6^bc^	8^abc^	69^c^
	Month 9	11^d^	7.9^bc^	67.8^de^
PPEFC(0.25% (w/w))	Initial day	11.5^bc^	8.5^a^	79^a^
	Month 3	11^d^	7.6^bc^	67.9^c^
	Month 6	11.3^cd^	7.6^bc^	68^c^
	Month 9	11.6^bc^	7.5^c^	68.1^c^
PPETX (1% (w/w))	Initial day	11.5^bc^	8.5^a^	79^a^
	Month 3	11.2^d^	7.7^bc^	68.2^c^
	Month 6	11.5^bc^	7.7^bc^	68.3^c^
	Month 9	11.8^bc^	7.7^bc^	68.1^c^
*CV* (%)		1.2	0.6	0.8
*P*-value	Storage period	<.0001	<.0001	<.0001
	Storage materials	<.0001	<.0001	<.0001
	Storage period ∗ storage materials	<.0001	0.02	<.0001

Means followed by the same letters within column are not significantly different (P > 0.05) by Tukey's test.

Vales are presented as Mean of three observations; PPB = Polypropylene bag; PB = PICS bag; GPB = Grainpro bag; PPBFC = Emmer wheat treated with 0.25% (w/w) filter cake and stored in Polypropylene bags; PPBTX = Emmer wheat treated with 1% (w/w) triplex and stored in Polypropylene bags.

The emmer wheat samples from PPBTX, PPBFC did not showed any significant difference in moisture content throughout the storage periods. The grains stored in PPB showed seed moisture content higher when relative humidity was peak in the storage environment. Under such conditions, PPB with larger size pores were unable to protect seeds from absorption of moisture from surrounding environment [14]. Relative humidity in the storage room is highly influenced by the weather pattern. Additionally, increase in moisture content of the grains stored in the storage bags is attributed to the moisture migration from the surrounding environment to the storage bag, as they are not moisture proof. Similarly, Khatri et al. [13], reported that wheat seed stored in earthen pot (is made of clay that is baked so that it becomes hard and used for seed/grain storage as farmers’ practice) recorded the highest moisture content than stored in hermetic bags (PB and GPD). In addition, Rahouma [11], reported that increasing storage periods of barley up to seven months in PPB gradually and significantly increased moisture content by 2.56%.

#### 3.3.2 Protein content

Protein content of stored emmer wheat samples was significantly (P ≤ 0.01) affected by interaction effect of storage materials and durations (Table 5). From the study finding observed that, as the storage period increased the protein content of emmer wheat decreased significantly. The initial protein content of 8.5% was observed, and then there was gradual decrease was find as storage duration increases from three to nine months. The least protein content (6.5%) was recorded in the emmer wheat samples stored in PPB for 9 months. However, no significant difference was observed in the protein contents of the emmer wheat stored in PB, GPB during the study period. Samples stored for 6 months in treatment run PPBTX, and PPBFC had showed the highest protein content than untreated PPB. The decrease in protein content of the emmer wheat samples stored in PPB may be attributed to the infestation of seeds by *R. dominica* in the storage period. The previous study [32] also reported similar observations as collaboration effect of storage durations and packaging bags had a substantial on protein content of stored wheat samples. In addition, depletion of protein along with other proximate and nutritional composition of wheat grains stored in farmers common storage is reported by Worku et al. [21], and same trend also reported in common bean by Mesele et al., [42]. The rapid reduction of protein content in extended storage periods stored in PPB is attributed to high moisture condition in this bag than the others during the 9 months. The proteolytic activity of the stored samples has been increased by higher moisture content in this bag. The increase in proteolytic activity leads to the breakage of polypeptide bonds in protein and converted to simple peptide chains that might have decreased the protein content of the sample [41]. Additionally, the reduction of protein content might be associated with infestation of emmer wheat samples by *R*. *dominica* during storage period.

#### 3.3.3 Carbohydrate content

The carbohydrate content of emmer wheat was strongly influenced (P ≤ 0.01) by the interaction effect of storage durations and materials used (Table 5). The emmer wheat samples stored in PPB for 9 months had observed for the lowest carbohydrate content (62%) whereas, samples stored in GPB for three months had the highest (78%) carbohydrate content. Emmer wheat samples stored in hermetic (PB and GPB) bags had not showed noticeable change in carbohydrate content. In a similar study Idah et al. [42] had reported that the carbohydrate content of tomato seeds also significantly decreased as storage periods increase. In addition, Tsado et al. [43] also reported that storage period had significant effect on rice carbohydrate content. The rapid reduction of carbohydrate content as storage period increased is attributed to the hydrolysis of available carbohydrates into sugars [41]. Samples from PPBFC, and PPBTX had showed a minor decrease in carbohydrate content during the enhanced storage periods but did not showed any significant difference (Table 5).

#### 3.3.4 Ash content

The major effect of both storage duration and storage bags used in this study showed a substantial (P ≤ 0.01) impact on the ash content of emmer wheat (Table 6). The ash content of emmer wheat reduced dramatically as the storage duration extended from three to nine months. Before storage, the original ash level of emmer wheat was 2.2 percent (Table 6). During nine months of storage, seeds housed in PPB had the lowest (1.9%) ash levels. The decrease in ash contents can be attributed to the emmer wheat respiration during storage. The amounts of carbohydrates, hydrogen, and oxygen in the seed drop during respiration. As a result, weight reduction is observed due to the carbon loss during respiration, and the ash content has dramatically decreased [47]. Adejumbo [45], on the other hand reported that, storage periods had no influence on wheat ash levels.

**Table 6:** Fat (%), fiber (%) and ash content (%) of emmer wheat as affected by storage periods, and materials.

Storage material	Storage durations	Ash (%)	Fiber (%)	Fat (%)
PPB	Initial day	2.2^b^	2.4^b^	3.8^a^
	Month 3	2^b^	2^c^	3.8^a^
	Month 6	2.1^b^	2^c^	3.6^a^
	Month 9	1.9^c^	2^c^	3.6^a^
PB	Initial day	2.2^b^	2.4^b^	3.8^a^
	Month 3	2^b^	2.3^b^	3.8^a^
	Month 6	2^b^	2.3^b^	3.8^a^
	Month 9	2^b^	2.3^b^	3.8^a^
GPB	Initial day	2.2^b^	2.4^b^	3.8^a^
	Month 3	2.1^b^	2.4^b^	3.8^a^
	Month 6	2^b^	2.4^b^	3.8^a^
	Month 9	2^b^	2.4^b^	3.8^a^
PPEFC(0.25% (w/w))	Initial day	2.2^b^	2.4^b^	3.8^a^
	Month 3	2.3^a^	2.5^a^	3.8^a^
	Month 6	2.3^a^	2.5^a^	3.7^a^
	Month 9	2.4^a^	2.5^a^	3.7^a^
PPETX (1% (w/w))	Initial day	2.2^b^	2.4^b^	3.8^a^
	Month 3	2.4^a^	2.5^a^	3.6^a^
	Month 6	2.4^a^	2.5^a^	3.6^a^
	Month 9	2.5^a^	2.6^a^	3.6^a^
CV (%)		1.2	1.4	2.6
*P*-value	Storage duration	<.0001	<.0001	0.24
	Storage material	0.1122	0.009	0.1
	Storage material∗duration	0.1	0.4759	0.4

Means followed by the same letters within column are not significantly different (P > 0.05) by Tukey's test.

Vales are presented as Mean of three observations; PPB = Polypropylene bag; PB = PICS bag; GPB = Grainpro bag; PPBFC = Emmer wheat treated with 0.25% (w/w) filter cake and stored in Polypropylene bags; PPBTX = Emmer wheat treated with 1% (w/w) triplex and stored in Polypropylene bags.

According to the result of the present study, the storage materials also influence the ash content. Significantly, highest (2.5%) ash content was recorded for emmer wheat samples from the treatment PPBTX. There is no significant difference between the ash content of samples from PPBFC and PPBTX. The TX and FC powders adhering to the seeds may be attributed to the increased the ash contents. This assumption is supported by the report of previous study [47], they reported that the highest ash content was recorded for diatomaceous earth (mainly composed of amorphous silicon dioxide) treated wheat samples. The TX and FC powders used in this study is also composed of silicon dioxide and other inorganic compounds [19]. Worku et al. [21] revealed in a similar study that wheat seeds treated with FC and TX had the greatest ash concentration.

#### 3.3.5 Fat content

Fat content is a significant factor in evaluating the wheat quality and appropriateness for various products, which can vary depending on seed storage conditions and time. Due to storage materials and times, the crude fat content of emmer wheat did not vary much (Table 6). The fat level of emmer wheat was 3.6 percent at the beginning of the trial and 3.8 percent at the conclusion. Similarly, Mesele et al. [39], found that neither storage procedures nor storage periods change the crude fat content of common bean.

#### 3.3.6 Fiber content

The main effects of storage duration and storage materials were significantly influenced the emmer wheat fibre content. According to ANOVA, the treatment interaction had no significant effect on fiber content (Table 6). The highest (2.6%) fiber content was recorded for emmer wheat from treatment PPBFC, and PPBTX for initial three months of storage whereas, the lowest (2 %) was observed for the samples stored for 9 months in PPB. In contrast to this results, the study conducted by Adejumbo [45], showed that fiber content of wheat is not affected by storage period whereas, El-Kady et al. [46] reported that the crude fiber content of rice is significantly affected by storage periods and materials studied. According to the findings of this study, the lowest fibre content of samples stored in PPB was attributed to the increasing of insects’ activity as storage periods progress, and resulting in vital nutritional components being consumed by storage pests, which leads in a decline in grain constituents.

## 4 Conclusions

In conclusion, the study results indicated that the traditional storage structure (polypropylene bags) resulted in seed quality loss (germination percentage, vigour index, speed of germination, TSW, BD, seed damage% and weight loss% of stored emmer wheat) and nutritional composition (protein, moisture, carbohydrate, fat, ash and fiber) as compared to hermetic (PICS &GPB) storage bags.

In general, the current study showed that seeds stored in GPB recorded the highest germination%, vigour and TSW by three months of storage. The highest speed of germination, protein and carbohydrate content by six months of storage. The hermetic bags (PB and GPB) and seeds treated and stored in PPB with FC and TX had showed the best performance on maintaining the seed quality and proximate composition of emmer wheat than PPB bags. Hence, replacing of PPB with GPB, PB by the farmers is a very important to maintain seed quality and proximate composition of emmer wheat in the study area. However, further investigations are suggested to conduct on the extended storage durations more than a year. Also the storage studies on the dehulled emmer wheat samples also suggesting. Moreover, the studies on the residual effect of filter cake and triplex in the human food should carry out in the further investigations.

## Declarations

### Author contribution statement

Bethlehem Melese, M. Sc: Conceived and designed the experiments; Performed the experiments; Analyzed and interpreted the data; Contributed reagents, materials, analysis tools or data; Wrote the paper.

Neela Satheesh, Ph. D; Solomon Workneh Fanta: Conceived and designed the experiments; Analyzed and interpreted the data; Contributed reagents, materials, analysis tools or data; Wrote the paper.

Zewdie Bishaw, Ph. D: Analyzed and interpreted the data; Contributed reagents, materials, analysis tools or data; Wrote the paper.

### Funding statement

This research did not receive any specific grant from funding agencies in the public, commercial, or not-for-profit sectors.

### Data availability statement

Data included in article/supp. material/referenced in article.

### Declaration of interest's statement

The authors declare no competing interests.

### Additional information

No additional information is available for this paper.
